# Oral Bran in Patients With Quiescent Ulcerative Colitis: Another Piece in the Diet Puzzle

**DOI:** 10.1093/crocol/otaa008

**Published:** 2020-02-13

**Authors:** Wael El-Matary

**Affiliations:** Department of Pediatrics, Max Rady College of Medicine, Rady Faculty of Health Sciences and Children’s Hospital Research Institute of Manitoba, Winnipeg, Manitoba, Canada

“What food should I eat?…. what food should I avoid?”. Very common questions that we all hear from our patients with inflammatory bowel disease (IBD). The role of diet in the pathogenesis of IBD through its effect on the intestinal microbiome is extremely important and has been the focus of basic and clinical research for years.^1^ The efficacy of several diets in the induction and maintaining remission of IBD has been under investigation. Nonetheless, only the exclusive enteral nutrition using elemental or polymeric formulas has been proven effective in induction of remission with a questionable value as a supplementary feed for maintenance of remission for Crohn’s disease.^2–4^ However, adherence to this diet can be challenging mainly because of exclusivity.^1^ A step toward improving adherence has been the introduction of a more tolerable diet, the Crohn’s disease exclusion diet, in a recent article by Levine et al.^5^ The Crohn’s disease exclusion diet includes a list of disallowed foods including gluten, yeast, dairy products, red meat (animal fat), processed meats, and products containing emulsifiers; it is a low-sugar but not low-carbohydrate diet with 18–20 g of fiber per day.^5^

On the other hand, the role of diet in managing ulcerative colitis (UC) has been under-investigated with less promising results as compared to Crohn’s disease. In an attempt to examine the role of diet in patients with UC, Nyman et al.^6^ randomized a group of adult patients with quiescent UC to either receive oral bran or low-fiber wheat products for 24 weeks. Oral bran seemed to increase fecal butyrate, lower serum low-density lipoprotein, and maintained no gastrointestinal symptoms. The number of relapses, however, was the same in both groups at the end of the follow-up period, which may be related to the sub-optimal concentration of fecal butyrate. Although the study is limited by the small sample size, high number of dropouts, and lack of studying associated changes in the intestinal microbiome, it adds an important piece of knowledge to the growing body of literature that investigates the role of diet in IBD.

## Notes

Funding: None.

## References

[1] El-Matary W . Enteral nutrition as a primary therapy of Crohn’s disease: the pediatric perspective. Nutr Clin Pract. 2009;24:91–97. 19244154 10.1177/0884533608329660

[2] Grogan JL , Casson DH, Terry A, et al. Enteral feeding therapy for newly diagnosed pediatric Crohn’s disease: a double-blind randomized controlled trial with two years follow-up. Inflamm Bowel Dis. 2012;18:246–253. 21425210 10.1002/ibd.21690

[3] Borrelli O , Cordischi L, Cirulli M, et al. Polymeric diet alone versus corticosteroids in the treatment of active pediatric Crohn’s disease: a randomized controlled open-label trial. Clin Gastroenterol Hepatol. 2006;4:744–753. 16682258 10.1016/j.cgh.2006.03.010

[4] El-Matary W , Otley A, Critch J, Abou-Setta AM. Enteral feeding therapy for maintaining remission in Crohn’s disease: a systematic review. JPEN J Parenter Enteral Nutr. 2017;41:550–561. 26645668 10.1177/0148607115621051

[5] Levine A , Wine E, Assa A, et al. Crohn’s disease exclusion diet plus partial enteral nutrition induces sustained remission in a randomized controlled trial. Gastroenterology. 2019;157:440–450.e8. 31170412 10.1053/j.gastro.2019.04.021

[6] Nyman M , Nguyen TD, Wikman O, Hjortswang H, Hallert C. Oat bran increased fecal butyrate and prevented gastrointestinal symptoms in patients with quiescent ulcerative colitis patients, a randomized controlled trial. Crohn’s & Colitis 360. 2020 (this issue). 10.1093/crocol/otaa005 PMC9802401 36777965

